# The role of gender in the decision to pursue a surgical career: A qualitative, interview-based study

**DOI:** 10.36834/cmej.69292

**Published:** 2020-08-06

**Authors:** Anita Acai, Kaushar Mahetaji, Susan E. Reid, Ranil R. Sonnadara

**Affiliations:** 1 McMaster University, Ontario, Canada; 2 University of Toronto, Ontario, Canada

## Abstract

**Background:**

Previous literature has explored the underrepresentation of women in surgery. However, this research has often been quantitative or limited by considering only the perspectives and experiences of women at more advanced career stages. Here, we use a qualitative methodology and a sample of women and men across the career continuum to identify the role that gender plays in the decision to pursue a surgical career.

**Methods:**

We audio-recorded and transcribed semi-structured interviews conducted with 12 women and 12 men ranging in their level of medical training from medical students to residents to staff surgeons. We used Braun and Clarke’s six-step approach to thematic analysis to analyze the data, maintaining trustworthiness and credibility by employing strategies including reflexivity and participant input.

**Results:**

Our findings suggested that the characteristics of surgery and early exposure to the profession served as important factors in participants’ decisions to pursue a surgical career. Although not explicitly mentioned by participants, each of these areas may implicitly be gendered. Gender-based factors explicitly mentioned by participants included the surgical lifestyle and experiences with gender discrimination, including sexual harassment. These factors were perceived as challenges that disproportionately affected women and needed to be overcome when pursuing a surgical career.

**Conclusions:**

Our findings suggest that gender is more likely to act as a barrier to a career in surgery than as a motivator, especially among women. This suggests a need for early experiences in the operating room and mentorship. Policy change promoting work-life integration and education to target gender discrimination is also recommended.

## Abstract (fr)

**Contexte:**

Des publications antérieures ont exploré la sous-représentation des femmes en chirurgie. Toutefois, cette recherche a souvent été quantitative ou limitée en considérant uniquement les perspectives et expériences de femmes à des stades plus avancés de leur carrière. Ici, nous utilisons une méthodologie qualitative et un échantillon de femmes et d’hommes a des stades différents de leur carrière pour établir le rôle que le genre joue dans la décision de poursuivre une carrière en chirurgie.

**Méthodes:**

Nous avons enregistré et transcrit des entrevues semi-structurées menées avec douze femmes et douze hommes, dont la formation médicale variait d’étudiants en médecine à résidents et à chirurgiens membres du personnel hospitalier. Nous avons utilisé l’analyse thématique en six étapes de Braun et Clarke pour analyser les données, conservant la fiabilité et la crédibilité en utilisant des stratégies qui comprenaient la réflexivité et les commentaires des participants.

**Résultats:**

Nos résultats suggèrent que les caractéristiques de la chirurgie et d’une exposition précoce à la profession constituaient des facteurs importants dans la décision des participants de poursuivre une carrière en chirurgie. Bien que ce n’était pas explicitement mentionné par les participants, chacun de ces domaines peut être implicitement sexospécifique. Les facteurs fondés sur le genre mentionnés explicitement par les participants comprenaient le mode de vie chirurgical et les expériences avec la discrimination fondée sur le sexe, y compris le harcèlement sexuel. Ces facteurs étaient perçus comme des obstacles qui touchaient de manière disproportionnée les femmes et qui devaient être surmontés dans la poursuite d’une carrière en chirurgie.

**Conclusions:**

Nos résultats suggèrent que le genre est plus susceptible d’agir comme un obstacle à une carrière en chirurgie que comme facteur de motivation, plus particulièrement chez les femmes. Cela suggère un besoin pour des expériences précoces dans la salle d’opération et le mentorat. Un changement dans la politique favorisant l’intégration travail-vie et une éducation pour cibler la discrimination fondée sur le genre est également recommandé.

## Introduction

Although women represent the majority of medical school graduates in Canada, their underrepresentation in surgery remains a persistent phenomenon.^1^^,^^2^ In 2015, women represented only 27% of all surgeons in Canada (Table 1) despite representing nearly 60% of medical school graduates since 2005.^3^^,^^4^ An even smaller number hold leadership positions^1^^,^^5^; for example, in 2019, only 12% of departmental chairs/heads of surgery in Canada were women. Moreover, women continue to be underrepresented in leadership positions even in specialties in which there are more women than men (e.g., obstetrics and gynecology [OB/GYN]), suggesting that there may be systemic barriers preventing women from moving into these roles.^5^

**Table 1:** Female and male surgeons in Canada, by specialty (2015)

Specialty	Female	Male
General surgery	503 (25.5%)	1,468 (74.5%)
Cardiac surgery	40 (11.3%)	314 (88.7%)
Neurosurgery	36 (11.0%)	292 (89.0%)
OB/GYN	1,232 (55.5%)	988 (44.5%)
Ophthalmology	295 (23.4%)	967 (76.6%)
Otolaryngology – head and neck surgery	151 (19.8%)	611 (80.2%)
Orthopedic surgery	182 (11.2%)	1,448 (88.8%)
Plastic surgery	136 (23.3%)	448 (76.7%)
Urology	77 (11.0%)	621 (89.0%)
Vascular surgery	27 (13.2%)	178 (86.8%)
**Total**	**2,680 (26.8%)**	**7,335 (73.2%)**

Gender is a significant determining factor in career selection, with fewer women interested in a surgical career than men.^6^^,^^7^ Data from the 2016 Canadian Resident Matching System showed that female^1^ medical school graduates were 40% less likely than males to rank a surgical specialty as their first-choice discipline.^8^ The fewest female applicants were found in specialties in which women have historically been the most underrepresented: cardiac surgery, neurosurgery, orthopaedic surgery, urology, and vascular surgery.^8^ Increasing the number of women in these specialties is an important step towards ensuring that the medical workforce is representative of the population it serves, which we believe may help improve access to healthcare. A greater representation of women in surgery may increase patient-centered care, particularly for women patients,^9^ and improve working conditions for the profession as a whole.^10^

In order to improve gender equity in surgery, a necessary first step is to understand the factors that contribute to individuals’ career choices. Existing literature on this topic has uncovered a variety of explanations for the lack of women in surgery, including the association of surgery with traits that are normatively masculine,^11^ a lack of role models and/or appropriate mentorship,^7^^,^^12^^-^^16^ experiences with sexual harassment and/or gender discrimination,^12^^,^^14^^,^^16^^,^^17^ and perceptions about the challenges of a surgical lifestyle and a lack of parental leave or childcare.^10^^,^^12^^,^^14^^,^^18^^,^^19^ However, a large number of the existing studies on gender in surgery are quantitative in nature, which may not fully capture the nuances of why and how these challenges are experienced. Furthermore, with the exception of work by Brown and colleagues,^10^ much of the work on gender in surgery focuses exclusively on the perspectives of women. While this is important to amplify and legitimize the voices of those who have historically been marginalized, we believe that gender equity can only be attained through a concerted effort from the surgical community as a whole, which includes individuals of all genders.

The goal of our research was to address the following research question: “What role does gender play in the decision to pursue a surgical career?” We approached this question using interviews that considered the experiences of both women and men across the career continuum, including medical students, residents, and staff surgeons.

## Methods

### Study design

We chose a qualitative, interview-based approach^20^ to our research as career choices are often complex and multi-faceted; thus, speaking directly with participants allowed us to develop a more nuanced understanding of how individuals decide upon a career in surgery and the role that gender plays in this process. We opted to use interviews over focus groups to respect the personal and potentially sensitive nature of some of the interview questions.^20^

### Participant recruitment

After receiving ethical approval from the Hamilton Integrated Research Ethics Board (HiREB-0629), we purposively^20^ generated an initial list of potential participants that included medical students, residents, and staff surgeons from two academic centres in Ontario, Canada. We included both women and men in our sample. We also attempted to recruit non-binary individuals; however, we were unable to identify any such individuals who were interested in or pursuing a career in surgery. Although participants were from Canadian centres, some participants’ backgrounds allowed them to speak to their experiences both within and outside of Canada, providing us with a rich data set. We reviewed our initial list of participants and filled any gaps using online department lists to ensure a sample that was representative of the various surgical specialties and levels of training/seniority within each participant category.

We contacted 30 potential participants via email and, if they agreed to participate, obtained written informed consent. We subsequently asked participants to complete a demographic questionnaire and scheduled an interview at a time and location of their choice.

### Data collection

We conducted semi-structured interviews with medical students, residents, and staff surgeons between February and June 2017. A sample interview guide is provided in Appendix A. This guide was adapted to medical students and residents; however, the topics covered were similar across all three participant groups. The questions within each interview guide were designed to be inductive and exploratory, allowing participants to share their experiences and opinions over the course of the interview.

A graduate student in health professions education (AA) and a research assistant (JW), both trained in qualitative research methods, conducted the majority of interviews in this study. On occasion, other research trainees also assisted with the interviews under the guidance of these researchers. Each interview lasted approximately 20 minutes (range: 10 to 50 minutes). No further themes arose after approximately 18 interviews; however, we continued sampling until we had an equal number of participants in each training category, and an equal number of women and men in our sample. The additional interviews helped to confirm themes and reinforce our conceptualizations of the data.

### Data analysis

We used Braun and Clarke’s six-step approach^21^ to thematic analysis to analyze the data. Thematic analysis is a flexible approach to qualitative data analysis that allows researchers to generate a rich, nuanced account of a phenomenon of interest.^21^ We began by familiarizing ourselves with the data by transcribing each interview and reading the resulting transcripts in their entirety several times. This was followed by the generation of initial codes, which identified features of the data that were relevant to our research question. We then grouped related codes into themes and generated a thematic map in order to further scrutinize the themes developed from the data. Once this process was complete, we defined and named the themes and used them to produce a written account of the data.

A graduate student in health professions education research (AA) and an undergraduate research trainee (KM) performed the data analysis. Both are non-MDs and therefore brought an outside perspective to this work. The research team corresponded frequently to discuss our conceptualizations of the data and to construct themes. We employed a number of strategies to help ensure the trustworthiness of the data, including reflexive journalling, documenting the analytic process, regular debriefing with more experienced clinical and non-clinical researchers, and seeking input from participants on the themes developed from the data.

## Results

Twenty-four individuals (12 women and 12 men) participated in the study. Participants came from two academic institutions, with one providing the majority (21) of participants. An equal number of medical students, surgical residents, and staff surgeons were included in our study. Collectively, these individuals represented seven surgical specialities. Participants had varying levels of training and experience in surgery. All medical students had experienced at least one surgical rotation and/or elective but had differing views on their likelihood of pursuing surgery. Five were clearly interested in a surgical career, two were clearly uninterested in a surgical career, and one was undecided and hesitant about a surgical career. Residents ranged from first to fourth year, while staff surgeons varied in their experience from newly appointed to approximately 20 years in clinical practice. A summary of the sample characteristics is provided in Table 2.

**Table 2:** Sample characteristics

	Medical Students (*n* = 8)	Residents (*n* = 8)	Staff Surgeons (*n* = 8)
**Gender**			
Women	5 (62.5%)	3 (37.5%)	4 (50.0%)
Men	3 (37.5%)	5 (62.5%)	4 (50.0%)
**Age**			
20-29	8 (100.0%)	6 (75.0%)	--
30-39	*--*	2 (25.0%)	1 (12.5%)
40-49	*--*	--	5 (62.5%)
50-59	*--*	--	2 (25.0%)
Year of Training*			
1	2 (25.0%)	1 (12.5%)	--
2	2 (25.0%)	2 (25.0%)	--
3	1 (12.5%)	2 (25.0%)	--
4	3 (37.5%)	3 (37.5%)	--
Academic Rank**			
Assistant	--	--	2 (25.0%)
Associate	--	--	6 (75.0%)
**Institution**			
Centre 1	6 (75.0%)	8 (100.0%)	7 (87.5%)
Centre 2	2 (25.0%)	--	1 (12.5%)

### Deciding on a career in surgery

Participants’ initial explanations of why they had chosen a surgical career did not explicitly make mention of gender-related factors. Instead, their responses focused on factors such as the characteristics of surgery and early exposure to the profession.

***Characteristics of surgery:*** Alignment between participants’ personal strengths and the characteristics of surgery was an important determining factor in their career choices. Participants emphasized the “doing” as opposed to “thinking” and “procedural” rather than “diagnostic” nature of surgery, which catered to individuals’ interests in a hands-on approach to medicine. One participant explained: “I really liked the hands-on aspect. I really like to be able to see the problem, diagnose the problem, and then fix it and see the results right away” (P10, F, Medical Student). Others highlighted the appeal of immediately visible outcomes, often leading to a sense of immediate gratification: “The first case I saw was a cancer case, so the patient walks into the room with cancer and they walk out, and it’s gone. That kind of immediacy … that’s what was really exciting to me” (P03, F, Resident).

Another appealing aspect of surgery was the ability to address a variety of complex health challenges as part of a team: “The teamwork is amazing. I just love it” (P16, F, Medical Student). Other described the “adrenaline rush” associated with high-acuity surgical cases, which some participants believed attracted “a very specific personality type. It’s the same type of people who would like certain high-adrenaline sports, and … become marathon runners” (P25, M, Staff). Participants also enjoyed the day-to-day variety inherent in a surgical career, both in terms of the cases encountered and the tasks performed.

***Early exposure****:* Early exposure to the profession was also an important factor in participants’ decision to pursue surgery. Participants listed classes with professors specialized in particular surgical specialties; electives, rotations, and observerships; and targeted programming as particularly influential in instigating interest in surgery. While positive experiences in surgery were influential in motivating trainees to pursue a surgical career, negative experiences could serve as a deterrent. For example, one participant described how his clinical clerkship made him feel that surgery was an intimidating environment: “Some of the trainees that were in that surgical specialty … gave me the sense that you needed to eat, breathe, and sleep only [surgery] in order to survive” (P21, M, Staff).

Previous academic and life experiences could also incite excitement in surgery. These experiences ranged from personal injuries to non-medical undergraduate and postgraduate backgrounds in construction, engineering, illustration, and sports. In other cases, however, participants’ interests directed them away from surgery. For example, a medical student with an interest in public health explained that he did not intend to pursue surgery because it “is so far away from everything that I am interested in” (P19, M, Medical Student).

***Gender-based factors:*** Although gender was not explicitly mentioned by participants when describing their motivations for pursuing surgery, most participants viewed it as a challenge that needed to be overcome when deciding on a surgical career. We elaborate on the various gender-based factors discussed by participants below.

***Lifestyle and becoming a parent****:* Issues around the surgical lifestyle and becoming a parent were the most prominent career-related challenges mentioned by participants. While participants did not always explicitly indicate what they meant by the term “surgical lifestyle,” common sentiments included the lengthy training period and long working hours that could sometimes preclude individuals from focusing on other areas of their lives (e.g., family, hobbies and interests). While both women and men acknowledged these challenges, they were seen to disproportionately affect women. One participant explained, “I think that at a cultural level, at an instinctive level, people still have biases for who’s supposed to be at home raising the children” (P07, M, Staff). Another remarked, “Gender doesn’t become a challenge until you actually become a mother. That’s where it becomes much more challenging” (P08, F, Staff). Participants described a variety of challenges related to becoming a parent including the challenges of pregnancy and breastfeeding while performing surgery; the social stigma associated with taking pregnancy and/or parental leave; challenges accessing childcare; fear of skill decline; and longer-term impacts of parenthood on career advancement, such as a longer time spent in residency training or fewer women in higher-ranking academic positions.

Lifestyle challenges were exacerbated when both spouses had demanding careers: “I think [in the past] the surgical lifestyle was based around the premise that you had a single-income family, where you had a spouse who was essentially at home … helping out with a lot of the … day-to-day work” (P17, M, Resident). Surgeons with spouses who had more flexible careers, on the other hand, were more likely to consider their partner an important source of support in maintaining a family life: “My husband decided not to work anymore, or at least not to work full-time, to be with our kids” (P08, F, Staff). Along similar lines, both the women and men in our sample acknowledged the importance of non-childbearing (i.e., “paternity”) leave in more equitably distributing childcare duties between genders. However, many participants found it hard to fully take advantage due to social stigma. For example, one participant recalled overhearing a senior surgeon say: “‘He’s losing money to raise a kid. … You should have your wife around to help you raise your kid.’” (P24, M, Resident).

***Stereotyping and gender discrimination:*** Additional challenges were related to stereotyping and gender discrimination, including sexual harassment, when interacting with colleagues and patients. Stereotyping among women was common, with many women reporting that they were often mistaken for nurses or medical students and could be met with resistance from nursing staff or allied health professionals when giving orders: “I find that they’re not necessarily coming to me with issues. They will come to the male juniors or … I’ll get a lot of resistance” (P04, F, Resident). Women participants also shared numerous examples of inappropriate comments made by patients or colleagues, for example: “‘I never thought I’d have … a beautiful young woman examine me like this’” (P11, F, Resident) or “‘If I was your dad, I wouldn’t have let … my daughter, go into general surgery’” (P16, F, Medical Student).

Gender discrimination was not exclusive to the women in our sample, although these challenges presented themselves differently in men than in women. A controversial finding was men in OB/GYN reporting on the difficulty of gaining clinical experience with sensitive procedures: “I think gender … played a big role in my learning. Just because patients didn’t want a male to come in and … do an exam” (P06, M, Medical Student). While some men felt that this was an example of gender discrimination that could affect their educational experiences in certain specialities, one might question the extent to which a woman’s autonomy to choose her care provider during sensitive procedures is actually discrimination or patient autonomy. Nonetheless, we chose to include this finding here given its prevalence in men’s responses. Some men also felt that they received harsher feedback compared to their women colleagues or were socially isolated because they did not conform to traditional, gender-based stereotypes. As described earlier, participants frequently commented on the social stigma that men choosing to take paternity leave could face from their colleagues, which served to reinforce traditional gender-based stereotypes of men as providers and careerists and women as stay-home caregivers.

***Specialty interests:*** The extent to which gender-based challenges affected participants’ career choices often depended on their specialty interests. Challenges tended to be attenuated in surgical specialties in which participants were better represented (e.g., for women, OB/GYN and general surgery) due to a greater number of role models and mentors. For example, one general surgery resident commented that the rumors she had heard about surgery being incompatible with a family were “exaggerated. … I don’t think it’s unmanageable to have a family. … I’ve seen quite a few … female surgeons who have children, who have families, who have hobbies, and are able to make all those things work” (P03, F, Resident). Specialties in which there were fewer women, on the other hand, were perceived to “deter females from being interested in it. … The attitude [is] that surgery [is] not a specialty for women” (P14, F, Staff).

***Positive effects of gender:*** It is important to note that gender did not always negatively influence participants’ decisions. In some cases, the effect was positive. For example, one resident felt that her identity as a surgeon was particularly important because “A lot of women really want a female urologist. … [They] don’t want to talk to men about [sensitive health issues]” (P11, F, Resident). Another participant felt that women bring the benefit of being “more compassionate [and] nurturing, [which] lends itself to caring in medicine” (P14, F, Staff). For other participants, the growing presence of women in surgery was seen to be making the field more humane, which would ultimately benefit everyone: “I think there’s been a lot of change because [women] have demanded that surgery … doesn’t kill your lifestyle, so that you can still be a human being and also do surgery” (P22, M, Resident).

## Discussion

This study explored the role that gender plays in the decision to pursue a surgical career using a sample of Canadian medical students, residents, and staff surgeons. While a small number of participants found their gender to be a motivating factor in their decision to pursue surgery, the majority of participants perceived gender as a challenge. Responses centred on the surgical lifestyle, becoming a parent, stereotyping, and gender discrimination, all of which were felt by participants to disproportionally affect women interested in a surgical career.

When discussing their main motivations for pursuing surgery, participants described an alignment between the characteristics of surgery and their personal interests, as well as early exposure to the profession. Although not explicitly mentioned by participants, each of these areas may implicitly be gendered. For example, scholars have argued that the surgical identity is constructed from a set of traits normatively identified as masculine.^11^ This view may be represented in our interview data, which focus on action-oriented characteristics of surgery such as the “doing” as opposed to “thinking” and “procedural” rather than “diagnostic.” It can also be seen in participants’ perceptions that one must “eat, breathe, and sleep” surgery in order to survive in the profession. While some women—particularly those who have selected surgery as a career—may find themselves aligned with these characteristics, others may find them alienating or intimidating. The “masculine” nature of surgery may also affect the extent to which women receive positive early exposure to the field, as there may be differences in how women and men participate in, and experience, surgical encounters in the operating room.^11^^,^^22^ Moreover, since there are currently more men than women in the field, it may also affect the extent to which new trainees are exposed to mentors who are women.

The specific manner in which the factors uncovered in this study intersected with one another to inform career decisions depended on participants’ circumstances, experiences, identities, and values. Although participants did not explicitly use the term “intersectionality” when describing their experiences, the notion that multiple identities and experiences can impact people in different and complex ways is important to consider yet has received very little attention in research on gender in surgery thus far.^23^ In our study, some participants were driven by their passion for surgery and appeared unphased by any gender-related challenges, despite being aware of and/or having experienced them. Others found their gender much more salient and “visible.” A number of participants reported an interest in surgery but were not fully comfortable with the perceived sacrifices, particularly around the surgical lifestyle and having children. These individuals were much less certain about the decision to go into surgery, and in some cases, had ruled out the profession entirely relatively early on in their training.

Prior research has shown that work-life integration is a primary concern when making career choices because it affects well-being and informs the sacrifices that individuals must make.^10^^,^^12^^,^^14^^,^^18^^,^^19^ A Norwegian study substantiated the impact of family life on career selection, finding that the more children a woman has, the lower her chance of becoming a specialist.^24^ Increasingly, however, lifestyle-related challenges are becoming important considerations for both women and men,^10^^,^^25^ and may help explain the decline or stagnancy in applications to more demanding specialities such as surgery.^12^^,^^26^ Our findings confirm the challenges associated with balancing the demands of a surgical career with raising a family and underscore the importance of policies around parental leave for both childbearing and non-childbearing parents. Not only must these policies exist on paper, but they must also be supported at all levels in order to be effective. Our study revealed gaps in this area, particularly with respect to non-childbearing (i.e., “paternity”) leave, as many participants found themselves supportive of, but unable to fully take advantage of this option due to social stigma and logistical challenges (e.g., scheduling).

It is also critical to pay attention to students’ early experiences in surgery. Consistent with other studies,^27^ our findings suggest that speciality decisions are often solidified before medical school. This is often rooted in students’ desires to be competitive for their speciality of choice and can thus shape future experiences, such as the electives they choose to take. Positive experiences in surgery through observerships or targeted outreach programs may be influential in cultivating an interest in surgery.^12^^,^^28^ Furthermore, the presence of positive role models in surgery may increase a junior physician’s likelihood of indicating interest in surgery.^29^ Role models, mentors, and sponsors provide the necessary support and guidance trainees require to navigate their professional lives, which may be especially important for women and other minorities.^7^^,^^12^^-^^16^ Observing a woman surgeon balance her career with family life implies that work-life integration is possible. More importantly, having a greater number of women role models in surgery helps create a culture in which it becomes appropriate and typical to discuss gender-related topics, such as family planning and parental leave.

Social challenges including poor interactions with patients, peers, and staff can also deter students from a career in surgery. Experiences of stereotyping, gender discrimination, and sexual harassment, can be particularly influential in the decision to pursue (or not to pursue) a career in surgery.^12^^,^^14^^,^^16^^,^^17^ Our findings attest that gender discrimination is common in surgery, particularly among women. This may be especially problematic among medical students who do not necessarily have the same agency as more experienced professionals to navigate these challenges.^30^ Prior research has also shown that abusive acts are not always recognized and dealt with appropriately, in part because of the “machismo” culture of surgery.^11^^,^^31^ Thus, policies, education, and cultural change are imperative in order to clearly underline that gender discrimination is not tolerated among and towards healthcare professionals.

## Limitations

This study was limited to the perspectives of participants from primarily one academic centre in Ontario, Canada. However, our sample contained three participants from a second academic institution who did not report any major institution-specific differences. We also did not uncover any major institution-specific differences from residents or staff surgeons who had completed earlier parts of their medical training at other institutions. Nonetheless, there remains room to expand the diversity of perspectives included in this, and other gender-related work. For example, our sample was not very ethnically diverse, nor did it include the perspectives of those who do not identify with binary conceptualizations of gender. Finally, our work considered the perspectives of only those at academic centres; in community centres, some women may hold leadership roles despite not having an advanced level of academic recognition.

## Conclusions

Given that nearly 60% of graduating physicians are women, medicine has made significant strides towards a more representative workforce. Yet, in surgery, women continue to represent less than 30% of the workforce and are not moving into leadership roles at the same rate as men. This suggests that the current “wait and with time, they shall come” strategy for attaining gender equity in the profession does not appear to be effective. In addition to addressing concerns around work-life integration and gender discrimination, surgical programs should actively target prospective applicants earlier in their training to ensure that they have a chance to experience the profession and benefit from exposure to role models and mentors occur before career decisions are made.

## Footnotes

1 Note that in this article we use the terms “female” and “male” (i.e., referring to sex) when citing sources or using quotes in which these terms are used; in all other cases, we use “women” and “men” to refer to gender.

## Appendix A: Sample interview guide (staff questions)

1. Can you give us a little background about yourself—e.g., surgical specialty, current position, location, etc.?
2. Can you talk a little bit about how you decided on a career in surgery? What were some of the factors that played into your decision?
3. One of the things we’re interested in learning more about is people’s perceptions of gender issues in surgery. From your perspective, why do you think that women are underrepresented in many surgical specialties?
4. In your own decision to apply to a surgical specialty, did you ever think about the role that gender could or would play? If yes, what were some of the things you thought about? [*Follow-up if the considerations appear to be barriers*] What encouraged you to still pursue a surgical career?
5. Thinking about your experience as a surgical trainee (i.e., during residency and/or fellowship) and now as a staff, did you experience any challenges/barriers that you feel were as a result of your gender? [*Follow-up*] How did you/do you deal with these barriers?
6. Again, thinking about your experience as a surgical trainee (i.e., during residency and/or fellowship) and now as a staff, did you observe any of your colleagues experiencing any challenges/barriers that you feel were as a result of their gender? [*Follow-up*] Have you ever taken any action to help mitigate any of these barriers? Please do not feel bad in answering no—we are just looking to get a sense of how both people have responded to these barriers. [*If yes*] What made you choose to take action?
7. Do you think that gender can ever be of benefit in a surgical career?
8. To what extent do you think that gender is of benefit in your specialty/institutional context (1 = not at all to 5 = it is a huge benefit), and why? To what extent do you think it is a challenge/barrier (1 = not at all to 5 = it is a huge challenge/barrier), and why? [*Follow-up*] Do you think that this would be the same or different in other surgical specialties?
9. Have you observed differences between departments or institutions in terms of the role that gender plays in surgical careers? [*Follow-up*] What factors do you think contribute to these contextual differences?
10. Do you think changes need to be made in order to improve experiences for women in surgery? [*If yes*] What is the most important change that you think needs to be made?
11. Much of the work on gender issues in surgery has focused on women because of the issue of underrepresentation. However, these studies often do not consider the experiences of other genders (e.g., men, those who do not identify with a particular gender, etc.). Are there any specific challenges that you feel these individuals might experience? [*If yes*] How might these be addressed?
12. Is there anything else that you would like to share with us on the topic of gender issues in surgery?
